# GrapHD: Graph-Based Hyperdimensional Memorization for Brain-Like Cognitive Learning

**DOI:** 10.3389/fnins.2022.757125

**Published:** 2022-02-04

**Authors:** Prathyush Poduval, Haleh Alimohamadi, Ali Zakeri, Farhad Imani, M. Hassan Najafi, Tony Givargis, Mohsen Imani

**Affiliations:** ^1^Indian Institute of Science, Bangalore, India; ^2^Department of Bioengineering, University of California, Los Angeles, Los Angeles, CA, United States; ^3^Department of Computer Science, University of California, Irvine, Irvine, CA, United States; ^4^Department of Mechanical Engineering, University of Connecticut, Storrs, CT, United States; ^5^School of Computing and Informatics, University of Louisiana, Lafayette, LA, United States

**Keywords:** brain-inspired computing, hyperdimensional computing (HDC), neuromorphic computing, machine leaning, memorization

## Abstract

Memorization is an essential functionality that enables today's machine learning algorithms to provide a high quality of learning and reasoning for each prediction. Memorization gives algorithms prior knowledge to keep the context and define confidence for their decision. Unfortunately, the existing deep learning algorithms have a weak and nontransparent notion of memorization. Brain-inspired HyperDimensional Computing (HDC) is introduced as a model of human memory. Therefore, it mimics several important functionalities of the brain memory by operating with a vector that is computationally tractable and mathematically rigorous in describing human cognition. In this manuscript, we introduce a brain-inspired system that represents HDC memorization capability over a graph of relations. We propose GrapHD, hyperdimensional memorization that represents graph-based information in high-dimensional space. GrapHD defines an encoding method representing complex graph structure while supporting both weighted and unweighted graphs. Our encoder spreads the information of all nodes and edges across into a full holistic representation so that no component is more responsible for storing any piece of information than another. Then, GrapHD defines several important cognitive functionalities over the encoded memory graph. These operations include memory reconstruction, information retrieval, graph matching, and shortest path. Our extensive evaluation shows that GrapHD: (1) significantly enhances learning capability by giving the notion of short/long term memorization to learning algorithms, (2) enables cognitive computing and reasoning over memorization graph, and (3) enables holographic brain-like computation with substantial robustness to noise and failure.

## 1. Introduction

We face increasing needs for efficient processing for diverse cognitive tasks using a vast volume of generated data (Bonomi et al., 2012; Chen and Lin, 2014). Therefore, there is a crucial need for scalable algorithms to learn and reason about each prediction on today's embedded devices. Particularly, memorization is an essential functionality that enables today's algorithms to provide a higher quality of learning and reason for each prediction or decision. Memorization gives learning and information processing algorithms prior knowledge to keep the context and define confidence. Unfortunately, existing deep learning algorithms have a weak and nontransparent notion of memorization. Although Recurrent Neural Network (RNNs) and Long Short-Term Memory networks (LSTMs) incorporate memorization, they are very difficult to train and still not fully transparent to explore on prior knowledge (Pascanu et al., 2013; Sodhani et al., 2020).

There are also other crucial challenges with existing memorization techniques. Running algorithms that incorporate memorization (e.g., RNNs and LSTMs) often results in extremely slow processing speed and high energy consumption or needs a large cluster of application-specific integrated chips (ASIC), e.g., deep learning on Google TPU (Jouppi et al., 2017). This computation complexity is beyond the capability of resource-constraint embedded devices. In addition, edge devices often rely on unreliable battery-based sources, fault-tolerant memory and logics, and noisy wireless communication (Van Kranenburg and Bassi, 2012; Lee and Lee, 2015). Unfortunately, today's algorithms require high precision training and have almost no robustness to such noise and failure. For example, the existing RNNs and LSTMs require high-precision floating-point representation to train (Courbariaux et al., 2014; Micikevicius et al., 2017). This makes these algorithms highly sensitive to possible noise or failure.

Recently, HyperDimensional Computing (HDC) has been introduced as an alternative computational model that mimics important brain functionalities toward high-efficiency and noise-tolerant computation (Kanerva, 2009). Unlike deep learning, HDC is a model of the Cerebellum cortex that biologically represents human memory. HDC is motivated by the observation that the cerebellum cortex operates on high-dimensional data representations (Zou et al., 2021a). In HDC, objects are thereby encoded with high-dimensional vectors, called *hypervectors*, which have thousands of elements (Rahimi et al., 2016b; Imani et al., 2017a, 2019b). HDC incorporates learning capability along with typical memory functions of storing/loading information. It mimics several important functionalities of the human memory model with vector operations which are computationally tractable and mathematically rigorous in describing human cognition. The natural memorization capability enables HDC to provide several advantages as compared to the conventional deep learning solutions: (1) HDC is suitable for on-device learning based on hardware acceleration due to its highly parallel nature (Li et al., 2016; Imani et al., 2017b; Hernández-Cano et al., 2021b), (2) hidden features of information can be well-exposed, thereby empowering both training and inference with the light-weight computation and a small number of iterations (Rahimi et al., 2016a; Mitrokhin et al., 2019), and (3) the hypervector representation inherently exhibits strong robustness against the noise and corrupted data (Imani et al., 2017b; Frady and Sommer, 2019; Frady et al., 2020).

HDC has been employed as a part of many applications, including genomics (Kim et al., 2020; Poduval et al., 2021a), signal processing (Karunaratne et al., 2021), robotics (Mitrokhin et al., 2019; Neubert et al., 2019), and sensor fusion (Räsänen and Saarinen, 2015), manufacturing (Chen et al., 2021), and detection/recognition tasks (Genssler and Amrouch, 2021). Although HDC is a memory model, existing algorithms do not well exploit HDC memorization capability. For example, in all existing HDC algorithms, memorization has a weak definition of information accumulation. However, as has been shown by neuroscientists, the brain has a more complex definition (O'reilly and Munakata, 2000; Hassabis et al., 2017; Chai et al., 2018). Our brain naturally clusters data and represents information as a graph structure, where objects and edges show the correlation between objects (Wiecki et al., 2015; Bassett and Sporns, 2017). Over time, these memory graphs get larger and more complex while the brain automatically forgets or approximates old information (Chien and Honey, 2020). In addition, the brain has a highly approximate but ultra-fast mechanism to retrieve information (Schacter and Slotnick, 2004). Although we can implement and represent a graph using existing database and graph processing systems (Lumsdaine et al., 2007; Sahu et al., 2017), such a system will be highly complex, costly, non-scalable, and far from biological systems.

Prior research works have already attempted to use vector symbolic architecture and hyperdimensional computing to represent and process graph knowledge. Work in Gayler (1998) exploited hyperdimensional computing for graph representation. This method is designed specifically for graph isomorphism and cannot support complex information extraction from graph representation. Work in Ma et al. (2018) used holographic reduced representation (HRR) to map graphs into high-dimensional space. However, this approach relies on external learning algorithms, i.e., neural networks, to extract knowledge from the graph. As a result, the HRR encoding mainly acts as a latent space encoding rather than a memory to store graph information. Another existing direction focused on finding a graph embedding in real vector space (Nickel et al., 2016). By characterizing the similarity of the nodes using some loss function, the dot product between vectors is proportional to the similarity, which can be used for knowledge learning. However, this approach is quite costly as it requires gradient descent. In addition, it is not suitable for graph memorization.

This paper defines a brain-inspired system that better represents HDC memorization capability. We introduce, GrapHD, a graph-based hyperdimensional system that encodes graphs into high-dimensional space and enables reasoning on that graph. We use high-dimensional vectors to holographically represent the nodes and memorize the graph. GrapHD enables several cognitive functionalities to operate over compressed encoded graph directly. The main contributions of the paper are listed as follow:

- GrapHD defines an encoding method that represents complex graph-based data structure into high-dimensional space. GrapHD supports a wide range of memory graphs, including weighted and unweighted graphs. Our encoder spreads the information of all nodes and edges across into a full holistic representation so that no component is more responsible for storing any piece of information than another. This brain-like holographic representation enables us to define highly efficient and robust cognitive operations over the encoded graph without accessing original data.
- Using this memorization model, we introduce an inference process that can be used to recover the graph information from graph hypervector. Our reconstruction process is iterative in nature and relies on noise prediction and cancellation. GrapHD defines several important cognitive functionalities over the encoded memory graph. These operations include memory reconstruction, information retrieval, graph matching, and shortest path.
- We propose the idea of graph refinement that increases the capacity of memorization. Inspired by human memorization, refinement iteratively checks and strengthens the already known knowledge. This ensures that the known information, e.g., graph nodes and their connections, is well memorized. We design a statistical model that mathematically defines the capacity of a hypervector to perform the tasks mentioned earlier.
- We also develop an in-memory architecture that operates as a tensor processor to accelerate GrapHD computation. Our architecture supports row-parallel NOR-based operation over binary vectors stored in non-volatile memory. Then, we extend it to enable complex operations and accelerate various GrapHD applications.

We evaluate GrapHD on a wide range of applications. Our evaluation shows that GrapHD memorization capability not only enhances the reasoning capability of existing machine learning systems but also improves the learning accuracy. For example, we offer GrapHD application to enhance the existing CNN model for the object detection task. Our results show that GrapHD achieves 3.8 × faster training and 1.7 × faster inference than RNN, while ensuring the same classification accuracy. Our evaluation also shows that our in-memory accelerator achieves 30.4 × faster and 61.5 × higher energy efficiency as compared to NVIDIA 1080 GPU. We also run GrapHD operations with the Nengo SPA module (Bekolay et al., 2014) to simulate how GrapHD can be adapted for Neuromorphic hardware, and use it to run our novel error correcting decoding process. This provides feasibility for GrapHD model to be used as the encoding for Neuromorphic models of the brain. An example where this can be used is SPAUN (Stewart et al., 2012), which is a spiking neural network which can perform multiple tasks without requiring re-wiring. SPAUN encodes the query information using Semantic Pointer Architecture which is then fed into the neural network, and GrapHD can be used as the encoder to better memorize relationships and correlations thus expanding the cognitive abilities of SPAUN. A few examples where a graph representation is natural is analyzing relationships in social media and knowledge graph representations (Pitas, 2016; Bi et al., 2019; Chian et al., 2021). Our model can also be used in graph constructions, where the Spiking Neural Network is supposed to construct graph representations of data, or to construct certain sub-graphs and clusters of an input graph based on certain rules and correlations.

## 2. Preliminary

**Hyperdimensional Computing:** The brain's circuits are massive in terms of numbers of neurons and synapses, suggesting that large circuits are fundamental to the brain's computing. Hyperdimensional computing (HDC) (Kanerva, 2009) explores this idea by looking at computing with ultra-wide words—that is, with very high-dimensional vectors or hypervectors. The fundamental units of computation in HDC are high dimensional representations of data known as “hypervectors,” which are constructed from raw signals using an encoding procedure. There exist a huge number of different, nearly orthogonal hypervectors with the dimensionality in the thousands (Kanerva, 1998; Ge and Parhi, 2020). This lets us combine such hypervectors into a new hypervector using well-defined vector space operations while keeping the information of the two with high probability. Hypervectors are holographic and (pseudo) random with i.i.d. components. A hypervector contains all the information combined and spread across all its components in a full holistic representation so that no element is more responsible for storing any piece of information than another.

In recent years, HDC or in general vector symbolic architecture has been employed in a range of applications, such as classification (Kanerva et al., 2000; Ge and Parhi, 2020; Zou et al., 2021b), activity recognition (Kim et al., 2018), biomedical signal processing (Moin et al., 2021), multimodal sensor fusion (Räsänen and Saarinen, 2015), distributed sensors (Kleyko and Osipov, 2014; Kleyko et al., 2018), voice recognition (Imani et al., 2017a), genomics (Kim et al., 2020; Poduval et al., 2021a), regression (Hernández-Cano et al., 2021a), and privacy (Hérnandez-Cano et al., 2021). For example, work in Simpkin et al. (2017) used vector symbolic architecture for representing and orchestrating complex decentralized workflows. Work in Rallapalli et al. (2019) developed a novel embedding mechanism for single graph nodes that co-learns graph structure and textual descriptions. A key HDC advantage is its training capability in one or few shots, where object categories are learned from one or few examples and in a single pass over the training data instead of many iterations. HDC has achieved comparable to higher accuracy compared to support vector machines (SVMs) (Rahimi et al., 2018; Imani et al., 2019b), gradient boosting (Imani et al., 2019c), and convolutional neural networks (CNNs) (Mitrokhin et al., 2019), as well as lower execution energy on embedded processors, compared to SVMs (Montagna et al., 2018), CNNs and long short-term memory (Imani et al., 2019b).

**Holographic Graph Representation:** There are existing research works focused on high-dimensional and holographic graph representation. Work in Gayler and Levy (2009) represented graphs in an HDC model by binding together vertices to represent edges and adding the vectors together. However, they specified only a single graph isomorphism problem that can be solved using their model, without specifying how their model can be generalized to solve additional problems. On the other hand, our model provides an end-to-end framework to perform various operations and problems that can be solved purely using HDC operations. Moreover, we also provide a novel method to recover the bundled information that is stored in the graph memory. This method uses the iterative noise canceling method, where the results at one iteration are used to guess the noise in the next iteration. Additionally, we also discuss a memory refinement process that can be used to expand the capacity of our hypervectors.

Work in Ma et al. (2018) used holographic reduced representation to map nodes into high-dimensional space. This mapping, which is based on HRR, aims to learn graph as latent space; thus, it does not explicitly memorize the graph. The inference process is done using a 2-layered neural network. As a result, this representation relies on a neural network and is primarily suited for learning. In contrast, in our method, the learning, inference, and memorization tasks can be performed using native HDC operations. This makes our architecture robust, efficient, and scalable and enables us to retrieve desired information more transparently.

Work in Nickel et al. (2016) introduced a method to find an embedding of a graph in a vector space. A graph embedding is usually a learning process to find vector representations of graphs such that the vectors representing two nodes are correlated based on the nodes' similarity within the graph. This representation is obtained using the gradient descent method, which is computationally costly. In addition, the vector generated as graph representation has very low dimensionality, e.g., *D* = 150. In contrast, we define it entirely differently as we do not find a graph embedding. Our solution chooses random hypervectors to represent each node and uses them to build up graph memory. Our model is able to represent information and perform cognitive and inference operations using orthogonality of random hypervectors. We only perform the tasks and decoding using native HDC operations like bundling, binding, and similarity search.

### 2.1. Hyperdimensional Primitives

Let us assume ${\vec{H}}_{1}$, ${\vec{H}}_{2}$ are two randomly generated hypervectors ($\vec{H}\in {\left\{-1,+1\right\}}^{D}$) and $\delta \left({\vec{H}}_{1},{\vec{H}}_{2}\right)\approx 0$ (δ is similarity metric defined below).

Binding (*) of two hypervectors ${\vec{H}}_{1}$ and ${\vec{H}}_{2}$ is done by component-wise multiplication (XOR in binary) and denoted as ${\vec{H}}_{1}$ * ${\vec{H}}_{2}$. The result of the operation is new hypervector that is dissimilar to its constituent vectors i.e., $\delta \left({\vec{H}}_{1}*{\vec{H}}_{2},{\vec{H}}_{1}\right)\approx 0$; thus binding is well suited for associating two hypervectors. Binding is used for variable-value association and, more generally, for mapping.

**Bundling (+)** operation is done via component-wise addition of hypervectors, denoted as ${\vec{H}}_{1}+{\vec{H}}_{2}$. The bundling is a memorization function that keeps the information of input data into a bundled vector. The bundled hypervectors preserves similarity to its component hypervectors, i.e., $\delta \left({\vec{H}}_{1}+{\vec{H}}_{2},{\vec{H}}_{1}\right)>>0$. Hence, the majority function is well suited for representing sets. Note that the vector that we get after bundling will have integer components, and will be an element of ℤ^*D*^ in general. We do not clip the values of the components back to ±1.

**Permutation (**ρ**)** operation, $\rho \left(\vec{H}\right)$, shuffles components of $\vec{H}$ with a random permutation of the *D* components of the hypervector, with ρ^*p*^ defined as ρ applied *p* times. The intriguing property of the permutation is that it creates a near-orthogonal and *reversible* hypervector to $\vec{H}$, i.e., $\delta \left(\rho^{p}\left(\vec{H}\right),\vec{H}\right)≃0$ when *p* ≠ 0 and $\rho^{-p}\left(\rho^{p}\left(\vec{H}\right)\right)=\vec{H}$. Thus, we can use it to represent *sequences* and *orders*.

**Reasoning** is done by measuring the similarity of hypervectors. We denote the similarity with $\delta \left({\vec{H}}_{1},{\vec{H}}_{2}\right)={\vec{H}}_{1}\cdot {\vec{H}}_{2}/D$, where ${\vec{H}}_{1}$ and ${\vec{H}}_{2}$ are two hypervectors, and · denotes the dot product.

### 2.2. Motivation and Overview

As neuroscientists have already shown, the human brain memorizes events as a sparse memory graph (Reijneveld et al., 2007; George, 2008; Tijms et al., 2013), where nodes are the objects/events, and the edges represent the correlation between them. The brain does reasoning and analogy by referring to this memory as prior knowledge. For example, as humans, when we see a set of events or objects repeatedly occurring together, these objects get a higher correlation in our graph memory. By referring to this memory, we can identify the correlated objects, make better decisions, and reason about them.

Although building up this graph is often easy, the main challenges are: (1) how to effectively represent this graph to enable highly efficient and robust brain-like memorization, and (2) how to perform information retrieval and reasoning on such representation. Unlike the existing graph processing algorithms that perform costly exact computations, brain memorization and cognitive computation are highly approximate and efficient.

**Figure 1:**
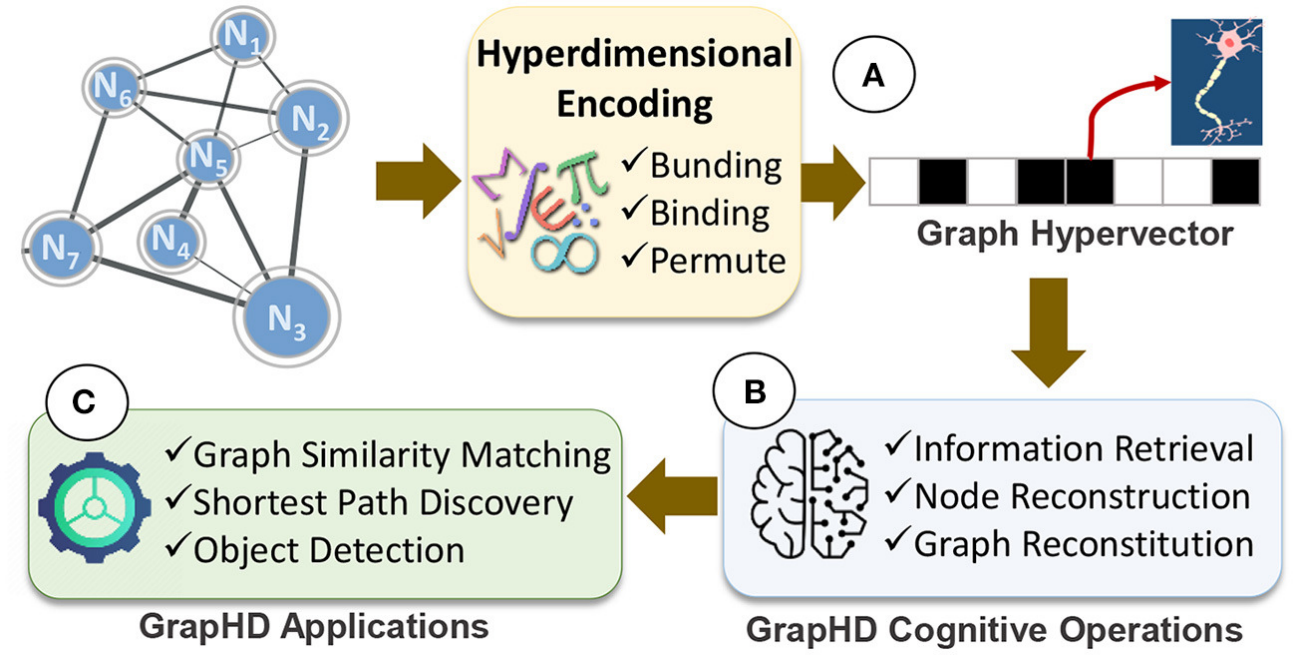
GrapHD overview: **(A)** hyperdimensional graph encoding into a hypervector, **(B)** GrapHD cognitive operations, and **(C)** GrapHD applications.

In this paper, we propose GrapHD, a hyperdimensional graph memory that enables robust, efficient, and holographic cognitive learning. Figure 1 shows an overview of GrapHD. GrapHD encodes various graph data into high-dimensional space (Figure 1A). The encoding is based on a well-defined set of mathematics introduced in Section 2.1. Our encoding represents a graph using a single hypervector, where each dimension represents a neuron. GrapHD enables a wide range of cognitive operations directly over the graph hypervector (Figure 1B). These cognitive operations extract information from the graph without explicit access to original nodes. We exploit these functionalities to enable several applications, including graph matching, shortest path, and object detection (Figure 1C).

## 3. Hyperdimensional Graph Representation

In this section, we explain how to represent graph structure in high-dimensional space. We exploit hyperdimensional mathematics, introduced in Section 2.1, to spread the graph information across the fully holistic high-dimensional representation. In this representation, no hypervector element is more responsible for storing any piece of information than another. Here, we explain how GrapHD encodes both weighted and unweighted graphs.

### 3.1. Unweighted Undirected Graphs

**Figure 2:**
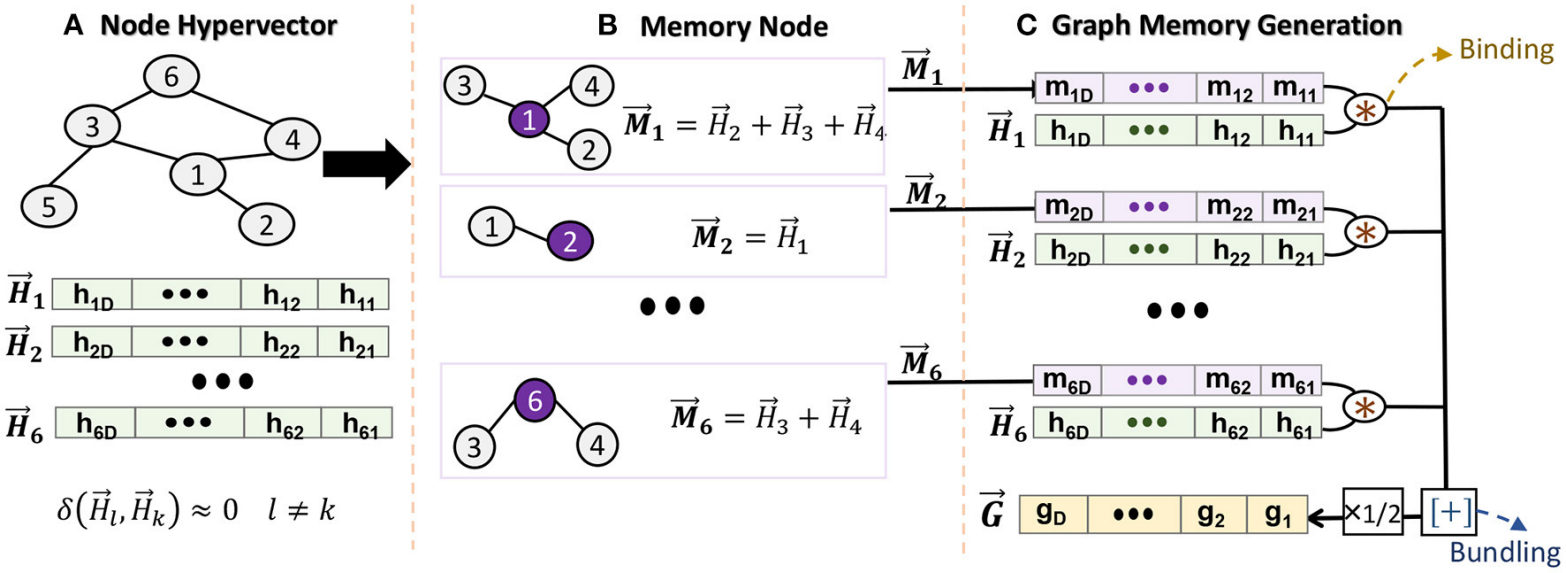
Graph memory encoding in GrapHD: **(A)** node hypervector generation, **(B)** creating a node memory, and **(C)** graph memory generation.

Figure 2 shows the functionality of GrapHD encoding representing unweighted graphs. We first assign a random hypervector ${\vec{H}}_{i}$ to each node in the graph (Figure 2A). Assuming a graph with *V* nodes and *E* edges, we generate $\left\{{\vec{H}}_{1},{\vec{H}}_{2},\cdots \;,{\vec{H}}_{V}\right\}$ as high-dimensional signature of nodes, where ${\vec{H}}_{i}$ is a *D*−dimensional hypervector whose components are randomly chosen from the set {−1, +1}. Due to random generation, the node hypervectors are nearly orthogonal: $\delta \left({\vec{H}}_{k},{\vec{H}}_{l}\right)≃0$ (*k* ≠ *l*), where δ denotes the similarity defined in Section 2.1. This non-zero similarity is the noise in our model which can result in misprediction. The role of noise in our model is further elaborated in Section 4.1.

We exploit the node hypervectors to create a memory for each node. The node memory needs to remember all connections that a particular node has to its neighbors (Figure 2B). For example, we construct the node *i* memory by accumulating all node hypervectors connected to it: ${\vec{M}}_{i}=\sum_{j}{\vec{H}}_{j}$, where *j* represents all the neighbors of node *i*. Thanks to HDC mathematics, the bundling keeps the information of all connections. For example, we can check if memory node *i* has connection to node *k* using: $\delta \left({\vec{M}}_{i},{\vec{H}}_{k}\right)$, where δ ≫ 0 and δ ≃ 0 show existence and non-existence, respectively. This is explained in Section 4.1 in detail.

After generating a memory for each node, we construct a single hypervector representing a graph. The graph memory should memorize the information of nodes and their connections. To this end, for each node, we associate the node and memory hypervectors, e.g., ${\vec{H}}_{i}*{\vec{M}}_{i}$ for node *i*. The bundling of all associated hypervectors generates a graph memory (Figure 2C):


$$\begin{array}{l} \vec{G}=\frac{1}{2}\left({\vec{H}}_{1}*{\vec{M}}_{1}+{\vec{H}}_{2}*{\vec{M}}_{2}+\cdots +{\vec{H}}_{V}*{\vec{M}}_{V}\right)\\ =\frac{1}{2}\overset{V}{\underset{i=1}{\sum }}{\vec{H}}_{i}*{\vec{M}}_{i} \end{array}$$

where the graph memory is a compressed, invertible, and transparent model. Note that we have introduced a factor of $\frac{1}{2}$ because if we expand the node memory, then ${\vec{H}}_{i}*{\vec{H}}_{j}$ and ${\vec{H}}_{j}*{\vec{H}}_{i}$ will be counted twice. Given the graph memory $\vec{G}$, we can reconstruct a local node memory using:


$${\vec{H}}_{i}*\vec{G}={\vec{M}}_{i}+noise\approx {\vec{M}}_{i}$$

where this approximate equality holds true because the HD vectors are randomly constructed; thus, they are nearly orthogonal. Once we have the node memory, we can check if nodes *j* and *i* are connected by calculating the similarity $R=\delta \left({\vec{H}}_{j},{\vec{M}}_{i}\right)$, where *R* is termed as the decision score. If there exists an edge between *i* and *j*, then *R* ~ 1. Otherwise, *R* ~ 0.

### 3.2. Unweighted, Directed Graphs

We use a similar encoding method as an undirected graph to build up each memory node. Since the graph is directed, each memory only bundles the connections out of the node. These memory nodes need to be combined to represent a graph. Unlike a undirected graph, the memory needs to preserve the sequence that nodes are connected together. Therefore, we construct the graph memory as: $\vec{G}=\sum_{i}^{n}{\vec{H}}_{i}*\rho {\vec{M}}_{i}$, where ρ is a permutation that permutes the node memory once, which is used to preserve the order of association. The edge between *i* and *j* is not treated the same as the edge between *j* and *i* because the permutation makes the binding a non-commutative operation. Therefore, compared to undirected graphs, there is no factor $\frac{1}{2}$ to construct the graph memory for directed graphs.

### 3.3. Weighted Graphs

**Figure 3:**
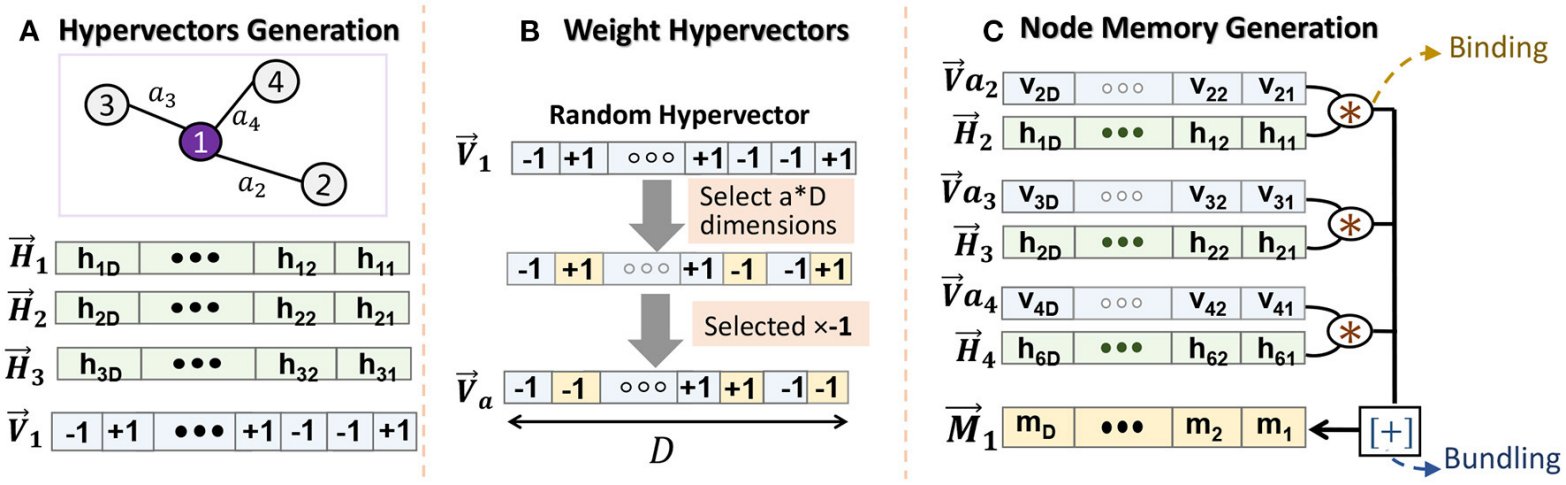
Node memory encoding in GrapHD for weighted graph: **(A)** node hypervector generation, **(B)** creating weight hypervectors, and **(C)** node memory generation.

In weighted graphs, the connection between nodes is represented using real values. To ensure holographic representation, our encoding needs to first represent those weights into hypervectors. Figure 3 shows GrapHD encoding for a node memory. Let us assume all weights in graph are normalized values [0, 1). we exploit stochastic representation to construct the vectors ${\vec{V}}_{a}$ for a real number *a* ∈ [0, 1). We generate ${\vec{V}}_{1}$ as a random hypervector representing a value 1 and exploit that to generate weight hypervectors. For example, we generate ${\vec{V}}_{a}$ by randomly choosing (1 − *a*) × *D* dimensions of ${\vec{V}}_{1}$, and multiplying them by −1. We define this evaluation of ${\vec{V}}_{a}$ as: $f\left({\vec{V}}_{a}\right)=\frac{\delta \left({\vec{V}}_{a},{\vec{V}}_{1}\right)+1}{2}=a$, where the final equality follows from the definition of ${\vec{V}}_{a}$. Although the randomness of weight hypervectors affects the robustness, the randomness makes this method undesirable when we look at the iterative method of decoding the node memory. The key problem is that slightly different values *a* will result in completely orthogonal vectors, which will eventually take up a lot of capacity. To avoid the above drawbacks, we generate ${\vec{V}}_{a}$ by flipping the components from (*a* × *D*)^*th*^ to the *D*^*th*^ component of ${\vec{V}}_{1}$. We note that we round *a* × *D* to the closest integer (Figure 3B). The evaluation function remains the same and provides the same result as before. This encoding is purely deterministic with respect to the weight value. Moreover, nearby values will generate correlated orthogonal vectors. As a result, we do not lose the capacity here and can represent a large set of weights.

Using stochastic weight representation, we can construct the node memory using ${\vec{M}}_{i}=\sum_{j}{\vec{V}}_{w_{ij}}*{\vec{H}}_{j}$ (Figure 3C). In this manner, we can store the weights in a holographic way such that the values of the weights do not bias the encoding. This is purely done using an end-to-end compatible HD framework.

### 3.4. Graph Memory Refinement

The brain is weak in one-pass memorization, as we often need multiple reviews of the same document to memorize the details. HDC also may not memorize every detail of a graph by single-time encoding and memorization (Gallistel and King, 2011; Ji et al., 2020). To ensure the information is well-memorized, HDC should look at a graph iteratively and strengthen nodes' information and connection. We name this process as *memory refinement*. In HDC, the model memorizes connections between nodes by bundling together hypervectors that represent different edges. However, these hypervectors are not perfectly orthogonal with each other. As a result, during the decision process, when the model calculates the similarity of a connection with the memory, the noise from the different connections can accumulate. This can lead to two possibilities: (1) the similarity of an existing node goes below a decision threshold, and (2) the similarity of a non-existing node goes above the threshold. These possibilities can result in misprediction of the connection.

The problem is that given a node vector *i*, we need to recognize which other nodes have vertices to node *i*. In other words, we need to check which memory nodes include the node *i* hypervector. This can be done by calculating the similarity of all memory nodes with node *i* hypervector:


$$\begin{array}{l} R_{ij}=\delta \left({\vec{M}}_{j},{\vec{H}}_{i}\right) \end{array}$$

where, *R*_*ij*_ is called the decision score. As described in Section 4.1, if *R*_*ij*_ is greater than *T* (called a decision threshold), then we conclude that ${\vec{H}}_{i}$ exists in ${\vec{M}}_{j}$. Our expectation is that all local memories which include node *i* should get a higher similarity than a threshold (*R* > *T*). The refinement procedure is done in multiple iterations. In each iteration, we chose a node *i* with node hypervector ${\vec{H}}_{i}$. Then, we go through the local memories of all the nodes *j* and perform the following update procedure


$$\begin{array}{l} {\vec{M}}_{j}\to {\vec{M}}_{j}+{\vec{H}}_{i}\text{ If }R_{ij}<T\;\text{but }j\;\text{and }i\;\text{share an edge}\\ {\vec{M}}_{j}\to {\vec{M}}_{j}-{\vec{H}}_{i}\text{ If }R_{ij}>T\;\text{but }j\;\text{and }i\;\text{do not share an edge} \end{array}$$

By this operation, we aim to strengthen the memory of the connections that are weakly memorized. However, refinement may result in some other connections being mispredicted. To prevent this, we perform memory refinement in an iterative manner until we converge into a final memory model. Note that the refined memory is an integer component hypervector.

Refinement is a process that is used in almost all other HD problems too, and is more commonly called Retraining. Almost all HD models require retraining to make them stronger in memory. Usually, retraining is implemented in models that use association search, where we match a query with multiple classes (For example, in classification tasks). Then, we subtract the query from the class with which it does not belong (with a factor proportional to similarity) and we add the query to the class with which it belongs if the similarity is not high enough. This results in a large separation between the similarity of the matching classes and mismatching classes with a query, which is the aim.

However, GraphHD uses a thresholding-based method to check if an node or edge exists (Based on Section 4.1 and 4.3, respectively) in the graph memory. For this, we need an alternative way of refinement which is different from the retraining used in the traditional context. This is why in our model we subtract or add the edge vector pairs to the graph memory based on whether the similarity is above or below the threshold, as described in the previous paragraph. Our aim here is not to differentiate between the classes, but to separate the signal distribution from the noise.

In Section 6.2, we show the impact of the memory refinement on increasing the hypervector capacity to memorize larger graphs.

## 4. Algorithms With GraphHD Representation

We perform several important cognitive functionalities over the memory graph to extract information or reason based on that. We discuss a few key capabilities which have a wide range of applications in robotic, genomics, signal processing, and machine learning. All tasks can be directly implemented over a single graph memory hypervector without storing original nodes or their connection. In other words, we will show how a single graph hypervector can answer several cognitive questions in a fast and efficient way. In the following, we demonstrate the algorithms only for an undirected unweighted graphs. However, the algorithms described can be extended to directed and weighted graphs without much difficulty. For directed graphs, we would have an additional step of applying an inverse permutation when reconstructing the node memory from graph memory, and using the permutation while checking existence of edge inside the graph memory. For the weighted graphs, we need to recover the weight of the edge using the similarity search, and then define a reasonable threshold for the similarity above which we can confidently conclude the edge actually exists inside the graph (and that the measure similarity is not the noise). We generate graphs randomly by first considering a fully connected graphs, and then deleting a random but uniformly chosen set of edges.

### 4.1. Information Retrieval

The main objective of information retrieval is to extract information about the edges connected to a node and the information associated with each node. We devise a statistical framework to study the errors and data recovery. Given the graph memory $\vec{G}$, we can use this to reconstruct the node memory. Using the node memory, we run inference to find the two main quantities—the nodes that share an edge with the current node and the information that has been associated with the current node via binding.

First, we consider the task of identifying whether a node *A* is connected to node *B* given the node memory ${\vec{M}}_{A}$. The node memory can be written as ${\vec{M}}_{A}=\sum_{i=1}^{d_{A}}{\vec{H}}_{i}$, where ${\vec{H}}_{i}$ is the hypervectors of all the nodes connected to *A* and *d*_*A*_ is the degree of the node *A*. If the hypervector of node *B* is given by ${\vec{H}}_{B}$, then we calculate the decision score *R* given by:


(1) $$\begin{array}{l} R=\delta \left({\vec{M}}_{A},{\vec{H}}_{B}\right)=\underset{Noise}{\underset{︸}{\overset{d_{A}}{\underset{i=1,i\neq B}{\sum }}\delta \left({\vec{H}}_{i},{\vec{H}}_{B}\right)}}+\underset{signal}{\underset{︸}{\delta \left({\vec{H}}_{B},{\vec{H}}_{B}\right)}} \end{array}$$

This is for the case that the node *B* is connected to node *A*. If not, then the signal term would become part of the noise term. The similarity between two random hypervectors can be written as $\delta \left({\vec{V}}_{1},{\vec{V}}_{2}\right)=\frac{1}{D}\left(\sum_{i=1}^{D}a_{i}\right)$ where *a*_*i*_ are random variables with values uniformly sampled from {−1, +1}. As a result by the central limit theorem, $\frac{1}{D}\left(\sum_{i=1}^{D}a_{i}\right)$ is a Gaussian distribution with mean 0 and standard deviation $\frac{1}{\sqrt{D}}$. Thus, by the central limit theorem again we get that


$$\overset{N}{\underset{i=1}{\sum }}\delta \left({\vec{V}}_{i},\vec{V}\right)\sim N\left(0,\sqrt{\frac{N}{D}}\right)$$

Where $\vec{V}$ and ${\vec{V}}_{i}$ are randomly chosen vectors, and N is an integer. Thus, in the case that *A* and *B* have an edge connecting them, then the decision score *R* follows a Gaussian $N\left(1,\sqrt{\left(d_{A}-1\right)/D}\right)$ distribution. When there is no edge between *A* and *B*, then *R* follows a Gaussian $N\left(0,\sqrt{\frac{d_{A}}{D}}\right)$ distribution. Using this, we can construct a theoretical Receiver operating characteristic (ROC) curve and then define a threshold value *T*. If *R* > *T*, then we can conclude that nodes *A* and *B* have an edge between them, and if *R* < *T* then we can conclude that there is no edge between *A* and *B*.

**Figure 4:**
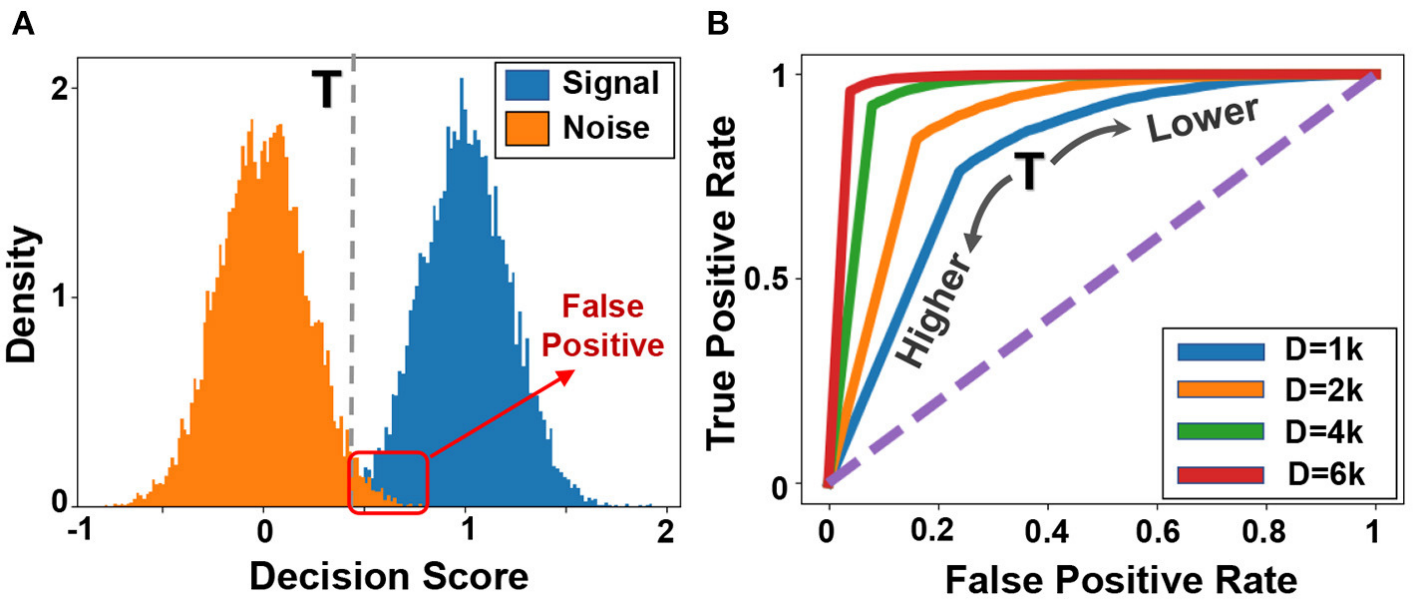
Information retrieval **(A)** Distribution of signal and noise during information retrieval, and **(B)** ROC curves for different dimensionalities.

Figure 4A shows the similarity distribution of existing patterns (blue color, called signal) and non-existing patterns (orange color, called noise) in the reference or memorized hypervector. Both signal and noise follow Gaussian distribution, where the spread is an effect of interference noise as shown in Equation 1. To identify the existence of a pattern, our goal is to put a threshold that can separate signal and noise distribution. Figure 4B shows the ROC curve indicating the impact of threshold value on true and false-positive rates. Ideally, we want the ROC curve to pass through the left-top corner, where true and false positive rates are 100 and 0%, respectively. The sharp turning point would represent the optimal scenario. However, the ROC would be less sharp if we decreased the dimensionality. For example, in *D* = 1*k*, signal and noise will have wider distribution; thus, the perfect true positive rate can only be obtained with a very high false-positive rate.

### 4.2. Node Memory Reconstruction

**Figure 5:**
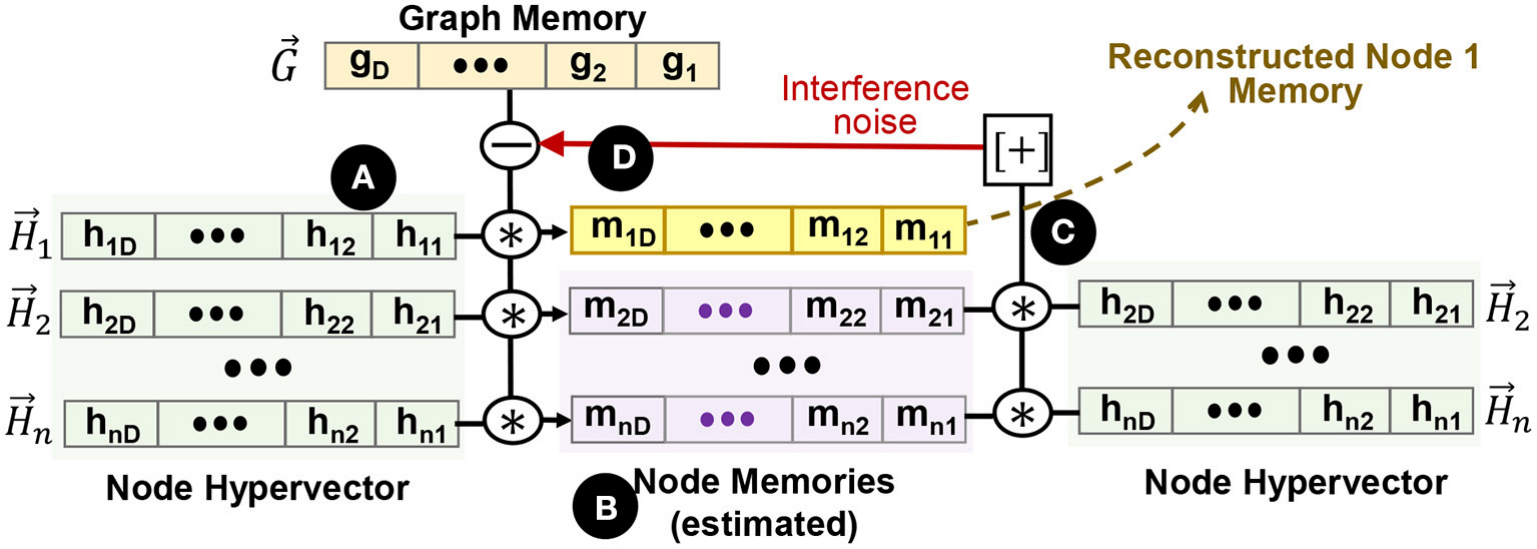
Node memory reconstruction. **(A)** node hypervectors, **(B)** estimated node memory based on node hypervectors, **(C)** cross-interference noise estimation, and **(D)** recursive noise cancellation in graph memory.

In this section, we discuss an iterative method to recover the node memory from the graph hypervector in an error-correcting way. The main idea is to first formulate a reasonable estimation of all node memories using the unbinding procedure. Then, we find a revised estimate for all the nodes by recursively canceling out the interference noise. Figure 5 shows GrapHD functionality for node memory reconstruction. Suppose we are given the graph memory hypervector $\vec{G}$. The first estimation of node memory *i* can be computed as, ${\vec{M}}_{i}^{\left(1\right)}$ (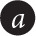):


(2) $$\begin{array}{l} {\vec{H}}_{i}*\vec{G}={\vec{M}}_{i}+\underset{≃0}{\underset{︸}{\underset{j\neq i}{\sum }{\vec{H}}_{i}*{\vec{H}}_{j}}}*{\vec{M}}_{j} \end{array}$$

Here, we use the fact that ${\vec{H}}_{i}*{\vec{H}}_{i}*{\vec{M}}_{i}={\vec{M}}_{i}$ because ${\vec{H}}_{i}$ is a bipolar vector. This equation gives us the first estimation of all node memories (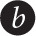), which is often noisy. The noise comes from the nearly orthogonal distribution of node hypervectors. Through an iterative process, we can start reducing the cross-interference noise (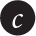). In each iteration, we find an estimation of memory nodes, ${\vec{M}}_{j}$ (*j* ≠ *i*), and deduct that noise from the next estimation. For example, we can recursively construct the following vectors (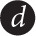):


(3) $$\begin{array}{l} {\vec{M}}_{i}^{\left(k+1\right)}={\vec{H}}_{i}*\left(\vec{G}-\underset{j\neq i}{\sum }{\vec{H}}_{j}*{\vec{M}}_{j}^{\left(k\right)}\right) \end{array}$$

(4) $$\begin{array}{l} ={\vec{H}}_{i}*\vec{G}-\underset{j\neq i}{\sum }{\vec{H}}_{i}*{\vec{H}}_{j}*{\vec{M}}_{j}^{\left(k\right)} \end{array}$$

The guess for the (*k* + 1)^*th*^ step is constructed by first subtracting the guess from the *k*^*th*^ step, which minimizes the error. For example, ${\vec{M}}_{i}^{2}$ is the revised estimate that we get from ${\vec{M}}_{i}^{1}$ (the first estimation) to cancel the noise. This process is repeated until we reach convergence. Section 6.4 explores the impact of different parameters on the quality of node memory reconstruction.

**Mathematical Capacity:** The accuracy of error reconstruction models depends primarily on two parameters: the number of edges of the graph, *n*, and the dimension of hypervectors, *D*. If there are more edges in the graph, then the cross-terms can contribute a higher value of noise in the iterative reconstruction step. A way to measure the noise can be done using the Signal Noise Ratio. Suppose that each node has on average *d* = *E*/*V* edges connecting to it. Then the node memory of a specific node is the sum of *d* different neighbor vertices of that node. Consider node *A*, with node memory ${\vec{M}}_{A}=\sum_{i=1}^{d}{\vec{H}}_{v_{i}}$, where *v*_*i*_ are all the nodes containing an edge to *A*. To check whether the node *B* has an edge with *A*, we calculate the similarity given by


$$\begin{array}{l} R=\delta \left({\vec{H}}_{B},{\vec{M}}_{A}\right)=\underset{Signal}{\underset{︸}{\delta \left({\vec{H}}_{B},{\vec{H}}_{B}\right)}}+\underset{Noise}{\underset{︸}{\underset{v_{i}\neq B}{\sum }\delta \left({\vec{H}}_{B},{\vec{H}}_{v_{i}}\right)}} \end{array}$$

The signal term is of magnitude 1, because every node vector is bipolar. Next, as we have demonstrated in Section 4.1, the noise term follows a gaussian distribution $N\left(0,\sqrt{\frac{d-1}{D}}\right)$. Thus, we can define the signal to noise ratio to be


$$\begin{array}{l} \text{SNR}=10\log\left(\frac{1}{\sqrt{\left(d-1\right)/D}}\right)\approx 5\log\frac{D}{d} \end{array}$$

As we can see, the increase of *D* decreases the noise, and we can decrease the noise by decreasing the average number of edges per vertices. Note that we assume the dimension *D* and the number of edges *E* are large to validate our approximation.

### 4.3. Graph Reconstruction

Here, we will discuss methods to reconstruct the whole graph given its memory hypervector $\vec{G}$. There are two main paths one can take for this: (1) follow the methods in Section 4.2 and first reconstruct the local node memory, and (2) use the methods of Section 4.1 to retrieve all the edges that are connected to the node via the node memory. We observe that the first technique can come with a large error rate. This is because the reconstruction of the node memory is not a binary classification process. Since we rely on convergence, the converged value of the node memory might have various errors that can make the node memory reconstruction vulnerable to error.

**Figure 6:**
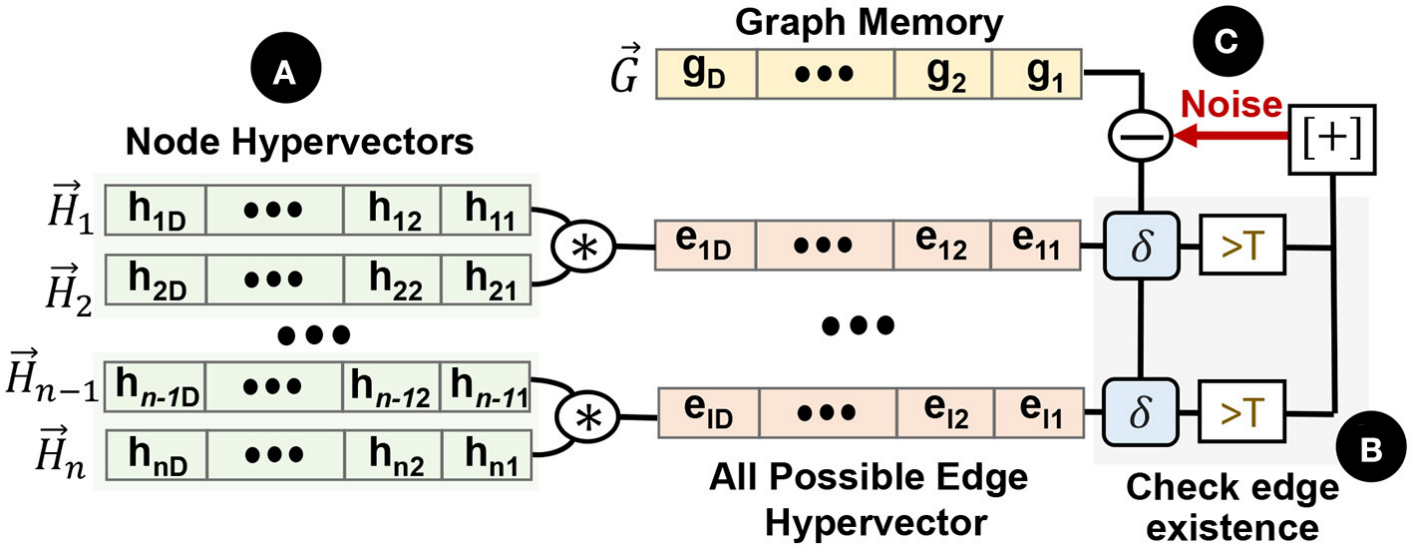
Graph memory reconstruction: **(A)** create all possible edge hypervectors, **(B)** checking the existence of each edge on the graph, **(C)** iterative noise cancellation.

In this paper, we present an iterative process to reconstruct the graph directly from the graph memory. We first define a function *f*(*A, B*) that checks the existence of an edge between nodes *A* and *B*. *f*(*A, B*) = 0 shows that there is no edge from node *A* to *B*, while *f*(*A, B*) = 1 indicates an edge. Figure 6 shows GrapHD functionality for graph memory reconstruction. In the first step, we generate a hypervector for all possible edges in the graph and initiate *f*^(1)^ = 0 for all edges. Then, we consider the existence of each edge (e.g., ${\vec{H}}_{A}*{\vec{H}}_{B}$) in the graph memory, $\vec{G}$ (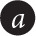). As we explained in Section 4.1, this existence can be computed by checking the similarity of the edge hypervector with graph memory (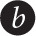). If the returned similarity value is larger than the threshold, we set *f*^(1)^ = 1. This is the inference process as described in 4.1. We repeat this process for all the nodes of the graph, and we construct the first estimation of the graph, ${\vec{G}}^{\left(1\right)}$.

Our goal is to enhance our estimation through an iterative noise cancellation method. Suppose there is an edge between *A* and *B* in *f*^(1)^(*A, B*), thus the noise vector is given by ${\vec{N}}_{AB}^{\left(1\right)}={\vec{G}}^{\left(1\right)}-{\vec{H}}_{A}*{\vec{H}}_{B}$. if there is no edge, the noise vector is simply ${\vec{N}}_{AB}^{\left(1\right)}={\vec{G}}^{\left(1\right)}$. In short, we can write this as (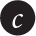):


$$\begin{array}{l} {\vec{N}}_{AB}^{\left(1\right)}={\vec{G}}^{\left(1\right)}-f^{\left(1\right)}\left(A,B\right)\left({\vec{H}}_{A}*{\vec{H}}_{B}\right) \end{array}$$

We construct the second estimation of the graph by initializing the function *f*^(2)^(*A, B*) = 0, and then checking whether the edge between *A* and *B* exists in the noise-corrected memory $\vec{M}-{\vec{N}}_{AB}^{\left(1\right)}$. If the result is positive, then we modify *f*^(2)^(*A, B*) = 1 and repeat the process for all pairs of nodes. This process is repeated iteratively as follows. Suppose we're given the *k*^*th*^ estimate of the graph *f*^(*k*)^. We initialize the graph representation *f*^(*k*+1)^ to 0. Then we use this to generate the graph memory ${\vec{G}}^{\left(k\right)}$ which corresponds to the graph *f*^(*k*)^(*A, B*). We calculate the noise for *A* and *B* as follows:


$$\begin{array}{l} {\vec{N}}_{AB}^{\left(k\right)}={\vec{G}}^{\left(k\right)}-f^{\left(k\right)}\left(A,B\right)\left({\vec{H}}_{A}*{\vec{H}}_{B}\right) \end{array}$$

We then check whether the edge from *A* to *B* exists inside the vector ${\vec{G}}^{\left(k\right)}-{\vec{N}}_{AB}^{\left(k\right)}$. If the answer is yes, we set *f*^(*k*+1)^(*A, B*) = 1 otherwise we set *f*^(*k*+1)^(*A, B*) = 0. We repeat the process until the convergence of the function *f*.

**Figure 7:**
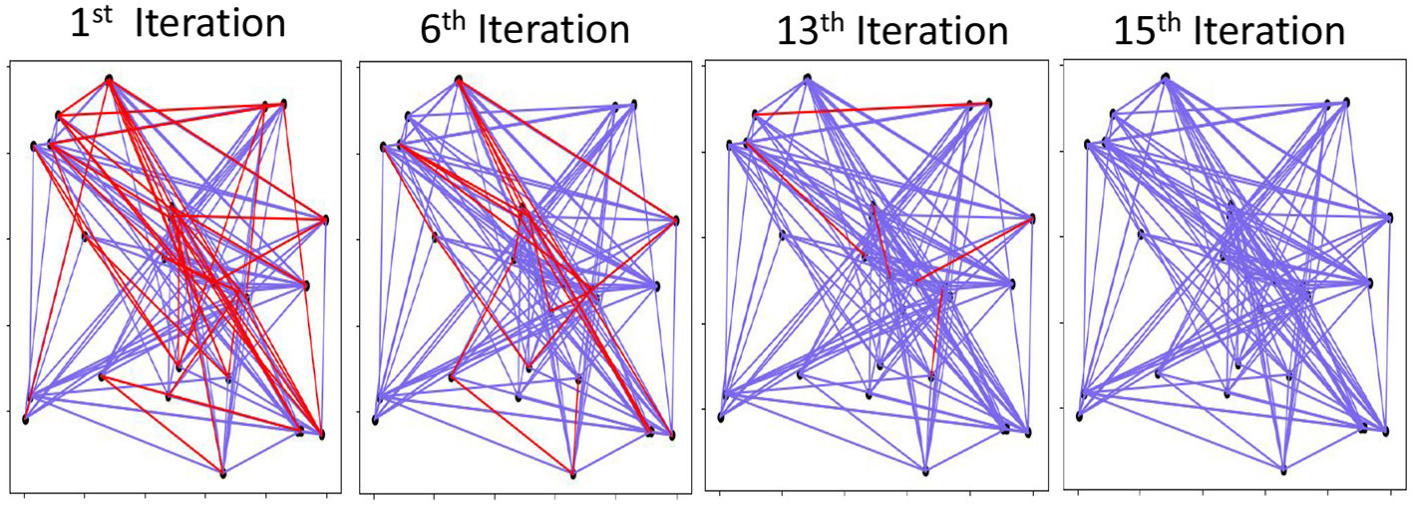
Visual graph reconstruction: red are mismatched edges and blue are existing edges. # Edges = 150, # Nodes = 30, and *D* = 3, 500.

Figure 7 shows a visual example of graph memory reconstruction during iterative noise cancellation. The results are shown for a graph with 30 nodes and 150 edges. The blue lines show the correct edges on the graph, while the red lines are edges in the actual graph but are not predicted by our graph reconstruction. Note that our method does not predict extra edges that are not a part of the graph. Our result shows that the initial graph reconstruction is approximate and cannot predict several existing edges. However, going further through iterative noise cancellation, we can get a higher accuracy by predicting more edges correctly. With 15 iterations, our technique can recover the entire graph accurately. Section 6.3 explores the impact of different parameters on GrapHD graph reconstruction.

### 4.4. Graph Matching

In this section, we formulate an algorithm to match two graphs directly using the HD framework. The aim here is to estimate the number of edges that occur in both graphs between the corresponding edges. One specific assumption we make about our model is that each node serves a specific function or specific memory. For example, in a cognitive model, we might have nodes that could represent items like cat, dog, animal, and pencil. The cat, dog, and animals would have edges among each other that represent the amount of correlation between them, while the pencil vertex would not be attached to any of them due to a lack of correlation with the other three items. This assumption is required to define the problem of graph matching appropriately. In cases where the node ordering does not matter, then matching two graphs has an additional component of finding a mapping between the vertices of two graphs which maximizes a similarity metric. However, by assuming the mapping of vertices to be fixed, our problem simplifies to finding how much the edges match.

Suppose we are given the graph memory $\vec{M},{\vec{M}}^{\prime }$ of two directed unweighted graphs *G* and *G*′. Then, using the method of Section 4.2, we can find the node memory ${\vec{M}}_{a},{\vec{M}}_{a}^{\prime }$ of node *a* of both the graphs *G*, *G*′, respectively. Our aim is to now compare these two graphs. To proceed, we find the difference of the node memory ${\vec{D}}_{a}={\vec{M}}_{a}-{\vec{M}}_{a}^{\prime }$ of node *a*. Now, we note that all the nodes that are connected to node *a* in both the graphs cancel out. Only the nodes that are connected to node *a* in exactly one of the graphs are present in the difference vector $\vec{D}$. We can write


$$\vec{D}=\overset{N}{\underset{i=1}{\sum }}{\left(-1\right)}^{n_{i}}{\vec{H}}_{i}$$

where *n*_*i*_ = 0 if ${\vec{H}}_{i}$ is connected to node *a* in *G* but not in *G*′, *n*_*i*_ = 1 if ${\vec{H}}_{i}$ is connected to the node in *G*′ but not in *G*. Here, *N* is the number of differences in the neighborhood of the node in both the graphs. That is, the number of nodes connected to the current node in exactly one of the graphs. Now, we use a statistical method to estimate the value of *N*. Each component of $\vec{D}$ is a sum of *N* random variables which take up values 1 or −1. Thus, each component of $\vec{D}$ goes as 2*B* − *N*, where *B* is a binomial distribution with *p* = 0.5 and *N* terms. The standard deviation of a binomial distribution is given by $\sigma_{B}=\sqrt{Npq}=\sqrt{N\times 0.5\times 0.5}=\sqrt{N}/2$. We can then use the method of moments to estimate *N*. Suppose *Y* = 2*B* − *N*. Then, we have 〈*Y*〉 = 2〈*B*〉 − *N* = *N* − *N* = 0. As a result, $\langle Y^{2}\rangle \langle =\sigma_{Y}$ is the standard deviation of *Y*. From the properties of standard deviation, $\sigma_{Y}=2\sigma_{B}=\sqrt{N}$. Thus, we can estimate *N* as 〈*Y*^2^〉. In Section 6.5, we show the capability of our proposed technique to enable efficient and parallel brain-like graph matching.

## 5. Neuromorphic Hardware Acceleration

GrapHD operations are highly parallel; thus, they can be accelerated on existing platforms. However, operating over long binary vectors could still be costly or non-optimized for CPU and GPU platforms. CPUs do not have enough resources for parallelism, and GPUs are more suitable for high-precision computations such as floating-point values (Halawani et al., 2021; Imani et al., 2021; Poduval et al., 2021b). To accelerate GrapHD, we develop a novel platform that naturally operates over long binary vectors. The capability of Non-Volatile Memories (NVMs) to act as both storage and a processing unit has encouraged us to use Processing In-Memory (PIM) platform for GrapHD acceleration. Since 2016, there have been several hardware accelerators for hyperdimensional computing based on processing in-memory technology. For example, work in Li et al. (2016), Imani et al. (2017b), and Imani et al. (2020) developed a novel PIM architecture accelerating associative search using content addressable memory. Work in Imani et al. (2019d) designed scalable PIM architecture to support encoding and scalable associative search. However, unlike existing hyperdimensional learning models, GrapHD is not based on association search. GrapHD performs computation using highly parallel (low-precision) arithmetic operation. This makes all existing hyperdimensional accelerators unable to accelerate GrapHD. GrapHD operations are mainly bitwise or low-precision vector-vector operations over long hypervectors. For example, binding is primarily based on XNOR operation between two vectors stored in different memory columns.

### 5.1. NOR-Based In-memory Computing

**Figure 8:**
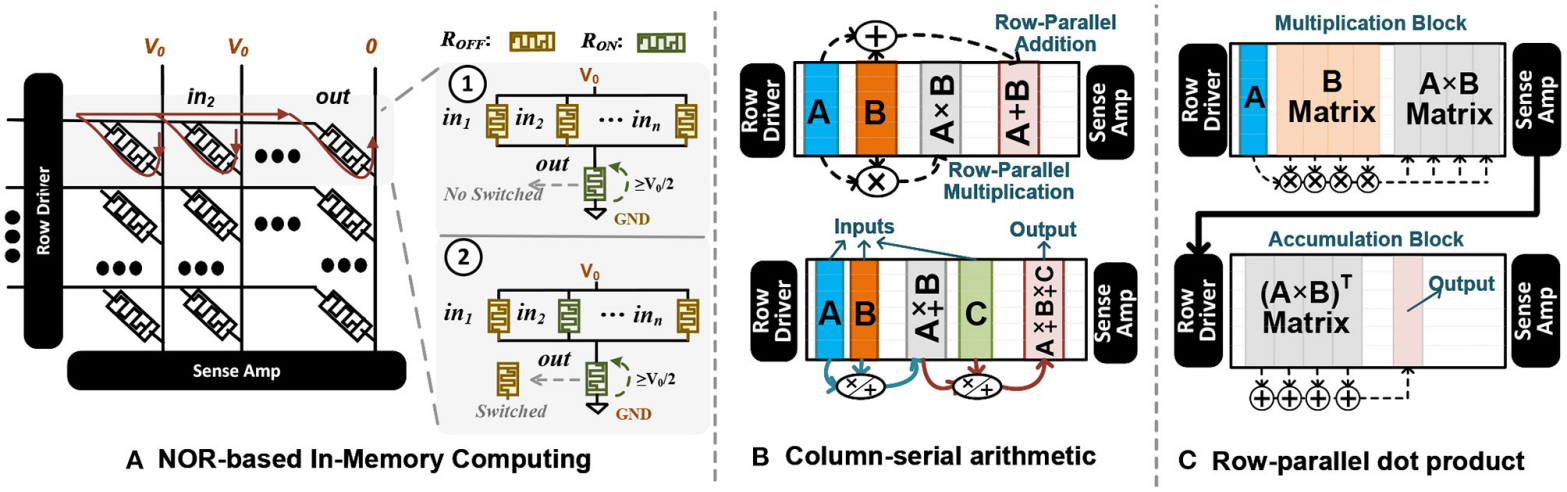
**(A)** NOR-based in-memory computing using switching characteristic of NVM devices. **(B)** row-parallel arithmetic operations, including addition and multiplication, and **(C)** row-parallel dot product operation.

In this paper, we develop DPIM that exploits the switching characteristic of memristor devices to internally perform the bitwise computation on the selected memory element without reading them out of array or using any sense amplifier. Figure 8A shows the structure of DPIM. DPIM exploits crossbar memory with single-bit NVM device and implements NOR operation in a row-parallel way among the selected memory columns (Imani et al., 2019a). In crossbar, each memristor device switches between two resistive states, *R*_*ON*_ (low resistive state, “1”) and *R*_*OFF*_ (high resistive state, “0”), whenever the voltage across the device exceeds a threshold (Biolek et al., 2021). This property can be exploited to implement NOR gate between the memory elements (Kvatinsky et al., 2014). Figure 8A also shows the NOR functionality on a single row of a crossbar memory. To execute NOR in a row, an execution voltage, *V*_0_, is applied at the *p* terminals of the inputs devices while the *p* terminal of the output memristor is grounded. If one or more input memristors are in a low resistance state (storing “1” value), the voltage across the output device will be *V*_0_, resulting in switching the output device to the high resistance stage (“0” value). However, if all input devices are in the high resistance stage, the voltage across the output device cannot switch the output device; thus, the output device keeps “1” value.

Since NOR is a universal logic gate, it can be used to implement other logic operations like addition and multiplication (Haj-Ali et al., 2018; Imani et al., 2019a). DPIM arithmetic operations are, in general, slower than the corresponding CMOS-based implementations. This is because memristor devices are slow in switching. However, this PIM architecture can provide significant speedup with massive parallelism. PIM can support addition and multiplications in parallel, irrespective of the number of rows. For example, to add values stored in different columns of memory, PIM takes the same amount of time to process the addition in a single row or all memory rows. Depending on the size of the operation, the computation takes a different time to execute. Let us assume the computation of *k* vertical vectors of *N*-bits with a length of *l*. When *k* ≤ *R*/*N* − *M*_*op*_, the execution time of addition and multiplication can be modeled as:


$$T_{op}=\underset{Crossbar\;Reuse}{\underset{︸}{\left(k-1\right)\times \left\lceil l/R\right\rceil }}\times T_{op}+w\times T_{write}^{D}$$

where *T*_*op*_ is the time of either fixed-point or floating-point arithmetic operations, *R* is the number of array rows, and 0 ≤ *w* ≤ *N* × *k* is the number of write operations.

### 5.2. DPIM Operations

In DPIM, at each time step, the main computation is a bitwise NOR operation between two columns of memory, storing two vectors. DPIM supports row-parallel computation, meaning that regardless of the number of rows, it takes the same amount of time to perform addition/multiplication. Figure 8B shows the functionality of DPIM performing row-parallel arithmetic operations. For any selected columns, DPIM computes a series of the NOR-based operations to implement bundling and binding. To perform computation among more than two vectors, the arithmetic operations are performing serially. For an example shown in Figure 8B, to perform arithmetic over three vectors, DPIM computes arithmetic between (*A* ± *B*), then the result is aggregated with the third vector (*A* ± *B* ± *C*).

DPIM only supports column-wise computation; thus, it cannot perform vector-matrix multiplication entirely in a single memory block. To address this, work in Imani et al. (2019a) proposed the idea of transposed vector-matrix multiplication that enables both multiplication and accumulation to happen using column-wise operations. This approach stores multiple copies of a transposed input vector (horizontal vector) in different memory rows. However, this method is slow and requires a large amount of reserved memory; thus, eliminating high-precision computation in a DPIM block.

To enable DPIM to perform accumulation in a row-parallel way, we propose a novel technique that enables multiplication and accumulation to be performed in two different blocks (Figure 8C). DPIM performs column-wise multiplication between the input vector and the matrix stored in memory. This multiplication is performed on the original data without transposing the input vector or matrix. To enable column-wise accumulation, our method writes the transposed multiplication results on the second block. To minimize the cost of data movement, we exploit the sense amplifier to perform row-parallel/bit-serial read operation of multiplication results and write them in the pipeline on the next memory block (accumulation block). This enables fast and efficient data transfer. Finally, we compute the vector-matrix multiplication by column-wise addition of the vectors in (*A* × *B*)^*T*^ matrix. In Section 6.8, we evaluate DPIM capability in accelerating different GrapHD applications.

## 6. Applications Evaluation

### 6.1. Experimental Setup

GrapHD has been implemented in both software and hardware co-module. In software, we verified GrapHD functionality by implementing it using Python on CPU. To ease the deployment on parallel platforms, we integrate GrapHD with PyTorch library. We optimized the PyTorch library to more effectively work with hypervectors as a common GrapHD data structure. We evaluated the framework on NVIDIA Jetson TX2, which has a CUDA-enabled GPGPU running with low-power profiles. We measure the latency of the learning procedure and the power consumption using the NVIDIA tegrastats utility.

We evaluate GrapHD functionality on multiple cognitive and learning tasks: (1) Graph memory and node memory reconstruction, (2) graph matching that checks the similarity of graph memories, (3) shortest path between nodes to reason about the relation and closeness of two memorized objects in the graph memory. (4) context-aware learning in object detection, where GrapHD is used as external memory to keep the relation between the objects occurring in different video frames.

For circuit-level simulation, we use HSPICE to measure the energy consumption and performance of DPIM in 28nm technology. The robustness of all proposed circuits, i.e., interconnect, has been verified by considering 10% process variations on the size and threshold voltage of transistors using 5,000 Monte Carlo simulations. DPIM works with any bipolar resistive technology, which is the most commonly used in existing NVMs. Here, we adopt a memristor device with a VTEAM model (Kvatinsky et al., 2015; Biolek et al., 2021). The memristor's model parameters are chosen to produce a switching delay of 1.1ns, a voltage pulse of 1V and 2V for RESET and SET operations to fit practical devices (Kvatinsky et al., 2014).

### 6.2. Graph Memory Refinement

**Figure 9:**
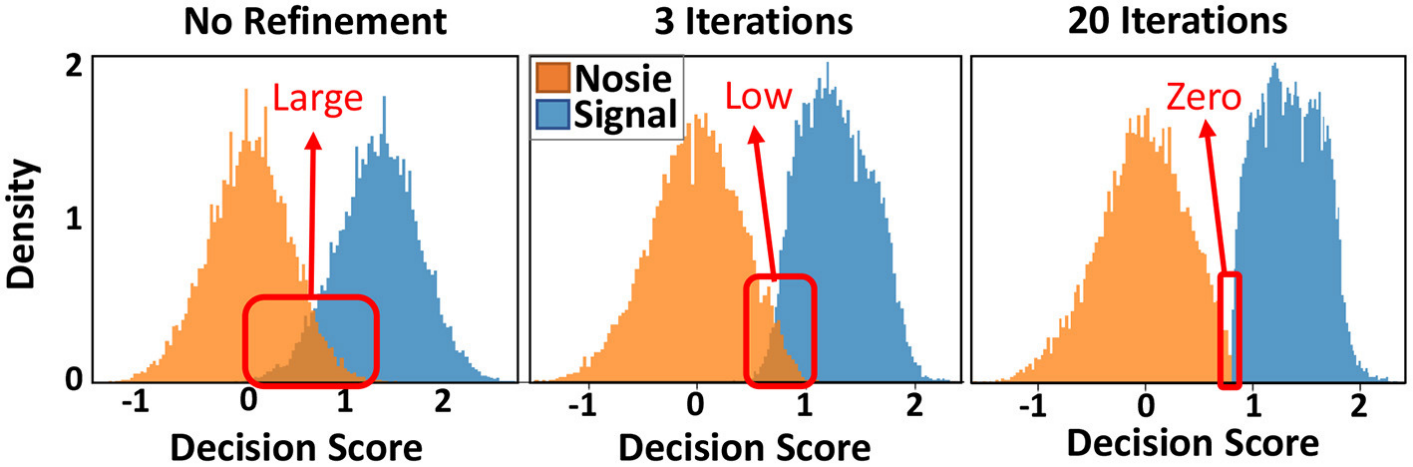
Distribution of existing and non-existing nodes in graph memory without and with an iterative graph memory refinement, shown for 100, 000 edges.

Figure 9 shows the similarity distribution of existing and non-existing patterns into graph hypervectors. The results are obtained for the initial (left) and the adjusted graph memory. As explained in Section 3.4, for perfect prediction and information retrieval, we would like to have no overlap between noise and signal distribution such that a threshold value can separate distributions. GrapHD memory refinement aims to iteratively increase the hypervector capacity and reduce the overlap between the signal and noise distribution. This would enable us to store large graphs in smaller dimensions. Graph refinement increases the similarity (decision score) of the existing patterns by recursively checking if the graph memory correctly memorizes them. For each misprediction (decision score lower than threshold *T* for existing patterns), we adjust the graph hypervector. As Figure 9 shows, the iterative graph refinement reduces the overlap between the noise and signal distribution until having zero overlaps in 20 iterations. This technique increases the capacity of a hypervector with fixed dimensionality to store a larger graph. In other words, this technique makes the ROC curve (Figure 4B) sharper, resulting in 100% true positive with 0% false-positive rates.

### 6.3. Graph Reconstruction

**Figure 10:**
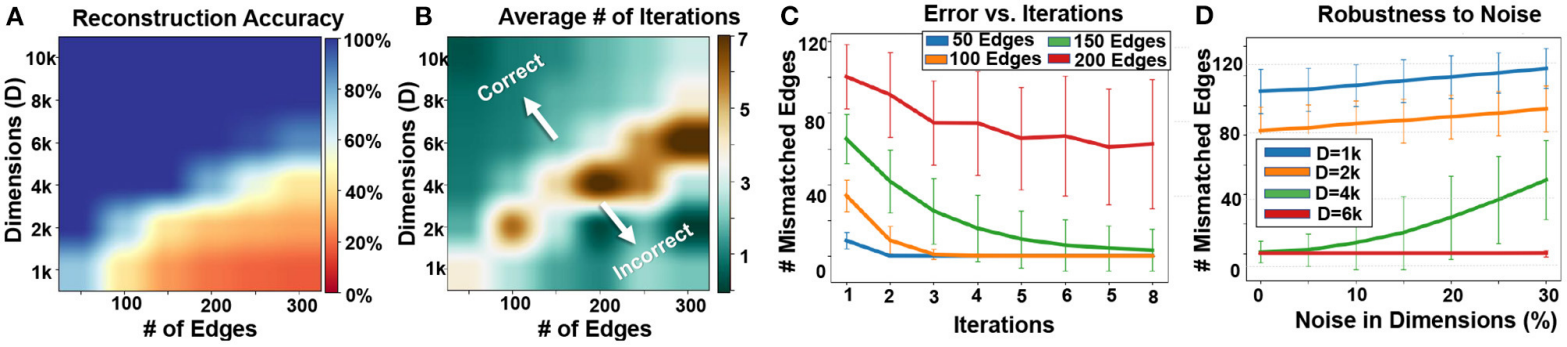
Graph reconstruction: **(A)** reconstruction accuracy, **(B)** required iterations vs. graph size and dimensions, **(C)** error rate v. iterations, and **(D)** robustness to noise (Shown over 1,000 trials).

Figure 10A shows the impact of hypervector dimensionality and the number of edges on the quality of information retrieval. Our results indicate a larger graph requires higher hypervector dimensionality to ensure full graph memorization. For example, a graph with 100 and 200 edges can be accurately stored in a graph hypervector with *D* = 4*k* and *D* = 6*k* dimensionality, respectively. Figure 10B shows the number of required iterations for data recovery. Our technique requires fewer iterations of noise cancellation when the dimensionality of a hypervector is larger than the number of edges that it can accurately store. On the other hand, when the dimensionality is much lower than the required value, our algorithm may still require a few iterations, but it would converge to a random solution. In summary, maximum iterations are required when the dimensionality is the lowest possible value that provides enough capacity to accurately recover the stored information.

Figure 10C also shows the number of mismatched edges during different noise cancellation iterations. Initially, our graph reconstruction comes with a large number of mismatched edges. This mismatch is larger for larger graph sizes. The error rate starts decreasing during our recursive error correction mechanism. When the size of the graph is within a capacity of a hypervector (*V* ≤ 150 for *D* = 4*k*, as shown in Figure 10A), our reconstruction will accurately recover the model. However, when the hypervector stores more patterns, our data recovery often diverges to a random graph (red line shown in Figure 10C). By increasing the number of vertices (and fixing the number of edges to 100), we find that the capacity is unchanged by the number of vertices for high dimensions. This could be because when we have a large enough number of vertices, then most of them will not be connected to any other vertices (due to a fixed number of edges). As a result, they will have 0 node memory and will contribute nothing to the graph memory, thus preserving capacity. The primary bottleneck is in generating orthogonal hypervectors that represent the nodes, so that in the decoding steps we do not make any false decisions. This is why at low dimensions we get a higher error, because the generated hypervectors for the nodes are not completely orthogonal to each other.

One of the main advantages of hyperdimensional representation is its high robustness to noise and failure. In GrapHD, hypervectors are random and holographic with i.i.d. components. Each hypervector stores the information across all its components so that no component is more responsible for storing any piece of information than another. This makes a hypervector robust against errors in its components. Figure 10D shows the impact of noise in dimensions on graph memory reconstruction. The results are reported when different percentages of hypervector dimensions are randomly dropped. Our representation provides inherent robustness to such noise, as the data can still be reconstructed when the dimensionality is large enough. For example, our method tolerates 10% random noise using *D* = 6*k* dimensions to represent a graph with 30 nodes and 150 edges.

### 6.4. Node Memory Reconstruction

**Figure 11:**
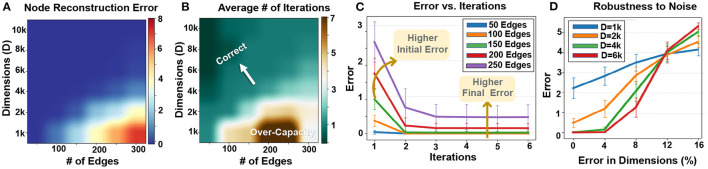
Node memory reconstruction: **(A)** error rate, **(B)** required iterations vs. graph size and dimensions, **(C)** error rate vs. iterations, and **(D)** robustness to noise.

Figure 11A shows the impact of graph size and hypervector dimension on node memory reconstruction error. Similar to graph reconstruction, the node reconstruction error depends on the graph size and dimensionality. A larger graph with more edges requires a higher dimensionality to ensure accurate node memory reconstruction. For example, for graphs with 100 and 200 nodes, our technique requires *D* = 2*k* and *D* = 4*k* to ensure 100% accurate node reconstruction. Note that using a hypervector with lower dimensionality to store a large graph could result in a quality loss during the information extraction. For example, using *D* = 2*k* to store a graph with 200 nodes reduces the chance of node memory reconstruction. Note that HDC is an approximate computational model. Therefore, it cannot theoretically ensure 100% data reconstruction. However, as our results show, in practice it is highly possible to get completely accurate reconstruction rate when your node memory is not loaded with more than its theoretical capacity.

Figure 11B also shows how the number of required iterations changes depending on the graph size and hypervector dimensionality. As expected, node reconstruction is faster when hypervector dimensionality is larger (in fixed graph size). The lower number of iterations comes from a low error rate and interference noises. Figure 11C shows the node memory reconstruction error for a hypervector with *D* = 4*k* dimensions that stores different graph sizes. The larger graph, the more iterations we need to cancel the noise. In addition, the noise is less likely to cancel out to decrease the error rate (as also shown in Figure 11A heatmap).

Similar to graph reconstruction, node reconstruction is inherently robust to noise and failure on random hypervector elements. Our evaluation in Figure 11D shows higher robustness in hypervectors with higher dimensionality. For example, hypervectors with *D* = 6*k* can tolerate a 5% error rate with no error. Even dropping more dimensions still has a small impact on the reconstruction error.

### 6.5. Graph Matching

**Figure 12:**
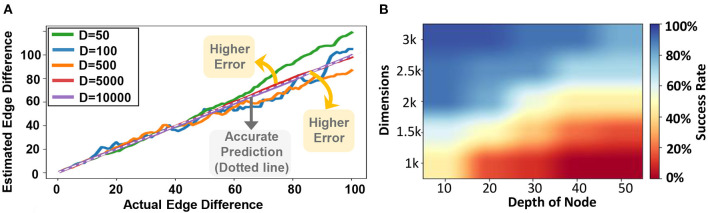
**(A)** Graph matching vs. dimensions: actual vs. estimated edge difference (For a single model) and **(B)** GrapHD success rate performing shortest path on an encoded graph (shown over 1,000 trials).

Figure 12 evaluates the quality of GrapHD for graph matching using hypervectors with different dimensions. For all evaluations, the graph size is assumed to be fixed (30 nodes and 150 edges). The x-axis in the graph shows the actual edge difference between the two graphs, while the y-axis shows our estimated node difference. Ideally, we expect to see a graph with a straight line (*y* = *x*), indicating that our estimation accurately matches the actual edge difference. However, graph matching comes with an error when the hypervector dimensionality is low. As our evaluation indicates, the estimated edge difference gets a higher error (becomes far from the diagonal line) when the dimensionality gets lower.

### 6.6. Shortest Path Between Nodes

In a graphical model of memory, identifying the context of information and making inferences often require identifying correlated nodes separated by certain distances in graphs. These are nodes that are not directly related; rather, they are related by a series of nodes that connect only to the next node. This is similar to how a reasoning process occurs; we start off with an observation connected to another memory. This memory would, in turn, lead to a connection with another memory through reasoning. This is equivalent to finding the path between two nodes and studying its connections in the graph.

We can use GrapHD algorithms to find a path between two nodes and find the shortest distance between them. Suppose we want to find the shortest path between two nodes *A* and *B* in a graph $\vec{G}$. First, we reconstruct all the local node memory for all nodes in the graph. Next, we maintain a distance value associated with all nodes, and this value is initialized to 0. This value will later be substituted with the step at which the node is encountered in the graph algorithm, which is also the distance of the node from *A*.

Here we explain our algorithm. In the first step, we consider the node *A* with node memory ${\vec{H}}_{A}$. Next, using the thresholding method from section 4.1, we find all the nodes that have an edge with *A*. These nodes are distance 1 away from *A*, and we assign a distance value of 1 to these nodes. Next, we consider the node memory of all the distance *d* = 1 nodes and add them together. Then we repeat the same process to find all nodes not encountered before that share an edge with the distance of *d* = 1 nodes. These nodes are a distance of *d* = 2 away. Suppose we have the set of all distance *d* = *n* nodes; we add up all their local memories. Then, we find the set of all nodes not encountered before, which share an edge of one of the distance *d* = *n* nodes. These nodes will be labeled with distance *d* = *n* + 1. The process is repeated until either the node *B* is encountered, until all the nodes are encountered, or no new nodes are encountered.

If the node *B* is never encountered when the process terminates, we conclude no path between the nodes *A* and *B*. If *B* is encountered, we begin finding out the exact path joining *A* and *B*. Suppose node *B* is at a distance *d* away from *A*. We consider the node memory of *B* and then find which one of the *d* − 1 distance nodes shares an edge with the node *B*. If there are multiple, we choose one of them randomly. Next, we consider the node memory of this *d* − 1 distance node. We find which of the *d* − 2 distance nodes share an edge with the *d* −1 distance node. If there are multiple, then we again chose one of the nodes arbitrarily. We continue this process recursively. After reaching the *d* − *k* distance node, we consider its node memory and find a *d* − *k* − 1 distance node that shares an edge with the distance *d* − *k* node. The process is continued until we reach the node *A*. Following the nodes back will allow us to find the shortest path that joins the nodes *A* and *B*.

In the evaluations, we simulate *d* disjoint random graphs, each with *V*_*av*_ = 21 vertices and *E*_*av*_ = 270 edges. In all these graphs, we chose one random node and labeled it as 1, 2, 3, 4, ⋯ , *d*. Next, we form an edge between all nodes. In this way, we construct a random graph that contains pairs of nodes with distances 1 to *d* in a controlled manner. In our evaluations, we chose *d* = 50 and the results are shown in Figure 12B. We see that as we increase the distance between the destination node and the starting node, the accuracy decreases drastically. This is because the number of edges that GrapHD searches through in each iteration increases exponentially with each step. As a result, the capacity eventually saturates if the graph is too big and if the distance is too large. On the other hand, increasing the dimension also increases the accuracy. This is expected since larger dimensions would increase the capacity of GrapHD, which allows storing a larger number of neighbors efficiently.

The shortest path detection has a natural interpretation in the case of weighted graph. In the weighted graph representation, the weights on edges become proportional to the similarity of the edge with memory. This results in a stochastic path finding algorithm, where the probability an edge is identified as being connected to the current node is proportional to the weight on edge. This has interpretation in the cognitive framework that the edge weight can be thought of as how strongly two nodes are correlated in the memory or how strongly the connection is memorized. The Human brain would form reasoning-based connections between two such objects depending on whether the two items in memory are strongly correlated. We can mimic a probabilistic path finding algorithm by using the current algorithm for the weighted graphs, which can mimic the reasoning process of the human brain. However, if we want to find a path independent of the weights, then we would need to store the graph using the unweighted encoding, and then the same algorithm would work as expected.

### 6.7. Object Detection

Based on the mathematical discussion in the paper (Section 4.1), we already showed the advantages that GrapHD for information retrieval, which is a key operation involved in traditional knowledge graph and relational learning benchmarks. Instead, in this work, we focus on a more advanced task that exploits knowledge graphs as a memorization model to enhance existing machine learning models. Our task also involves operations and computations that are not in high-dimension. Particularly, we evaluate GrapHD capability to help existing object detection algorithms. Deep learning models have already been used for highly accurate object detection (Ren et al., 2015). Particularly, convolutions neural networks (CNNs) showed promising results in extracting information from image and video data. However, CNN has a weak notion of time; thus, their predictions might be non-sense or out of context, e.g., predicting a moon as a light in videos taken from the sky.

GrapHD is a memorization model that can be used beside any learning algorithm. To eliminate these miss-predictions, CNNs need to keep the context by associating the objects during the training and inference phase. We exploit GrapHD to memorize the relation of objects as a memory graph. GrapHD assigns strong weights between objects that are more likely to happen together in a video frame. For every prediction, CNN predicts all objects that have been seen in a frame. Next, GrapHD encodes the objects into high-dimensional space and checks the graph memory to see a possible correlation of these items (i.e., the distance or existence of edges in a graph memory). This enables CNNs to provide more accurate decisions and also the capability to reason about the prediction based on prior knowledge. To get the maximum benefit from GrapHD, the learning and memorization models need to be integrated. In other words, both CNN and GrapHD models need to be updated using the same procedure and rules. In our study, the GrapHD is placed as a dynamic memory beside the CNN. For each given train data, the data is processed using both CNN and GrapHD. At first, CNN operates over the data to make a prediction. Next, GrapHD look at the CNN prediction and accordingly gives a new loss term to the CNN in order to get updated. This loss represents how far the CNN prediction was compared to a GrapHD memorization prediction.

**Figure 13:**
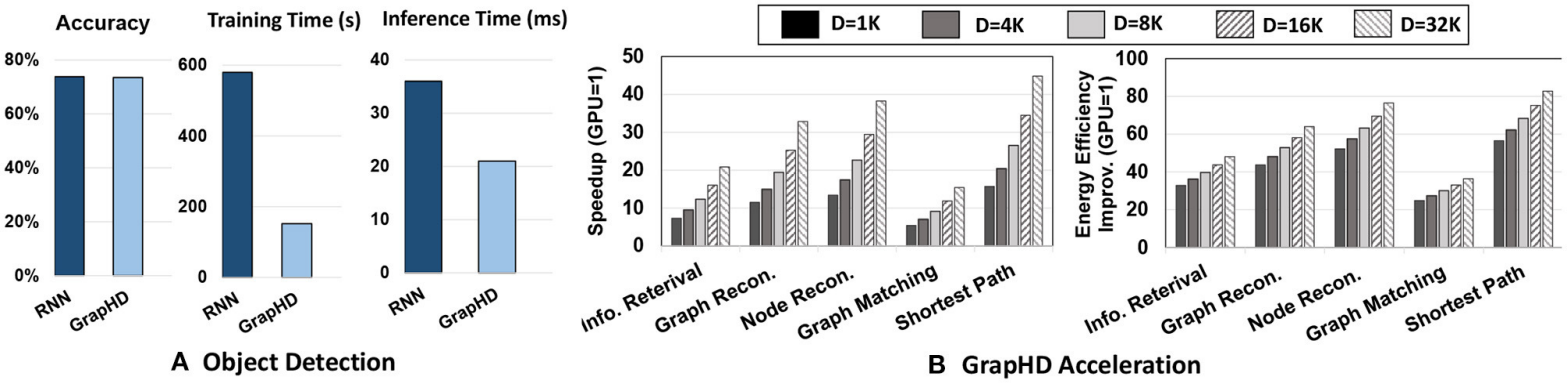
**(A)** GrapHD vs. RNN for object detection: Accuracy and efficiency. **(B)** DPIM speedup and energy efficiency running different GrapHD operations over GPU.

Figure 13A shows the accuracy and efficiency of CNN enhanced with GrapHD and recurrent neural networks (RNNs) for object detection task (Karpathy and Fei-Fei, 2015). The results are reported over the Microsoft COCO object detection dataset (Lin et al., 2014). Work in Karpathy and Fei-Fei (2015); Kousik et al. (2021) integrated CNN and RNN in series, thus providing memorization capability for CNN in making a prediction. The results are reported for networks running on NVIDIA Jetson TX2, an embedded processor. Our evaluation shows that CNN enhanced with GrapHD can provide the same accuracy as the RNN network. However, our method can provide significantly higher computation efficiency. Our solution enables parallel construction of CNN and GrapHD model, thus enabling parallel training. Our evaluation shows that GrapHD achieves 3.8× faster training and 1.7× faster inference than RNN while ensuring the same classification accuracy. Note that GrapHD provides a higher capability for reasoning, as it has direct access to the transparent memorized values.

### 6.8. Hardware Acceleration

As we explained in Section 5, GrapHD applications can be accelerated on parallel platforms. Here, we study the capability of the proposed DPIM architecture in accelerating GrapHD applications. Figure 13B shows the performance and energy efficiency of DPIM running different GrapHD applications. The results are reported for a large graph with 1,000 nodes that have been mapped to a hypervector with different dimensions. All results are reported respective to NVIDIA GTX 1080 GPU when GPU runs multiple queries to ensure maximum resource utilization. Our results indicate that DPIM provides higher speedup and energy efficiency as compared to GPU regardless of the dimensionality and GrapHD operation. For example, DPIM achieves 10.6× faster and 42.0× higher energy efficiency than GPU with *D* = 1*K* dimensions. DPIM efficiency depends on two factors: **(1) Application:** operations required by GrapHD applications. DPIM provides higher benefits for applications that require lower precision arithmetic. This is due to a linear and quadratic increase in DPIM bundling and binding time in respect to bit-precision. For example, GrapHD during graph and node reconstruction operates over low precision hypervectors, thus providing higher computation efficiency over GPU. **(2) Dimensionality:** DPIM efficiency increases with the hypervector dimensionality. This efficiency comes from DPIM capability to support fast and row-parallel operations and also address data movement issues by eliminating costly data access to off-chip memory. Our results indicate that DPIM provides significantly higher performance speedup for graphs with higher dimensionality. For example, GrapHD using *D* = 16*K* and *D* = 32*K* dimensions provide on average 23.1 × and 30.4 × faster computation compared to GPU. In terms of energy efficiency, DPIM efficiency has a lower relation to dimensionality as both DPIM and GPU will require the same number of operations. The slight improvement in DPIM energy efficiency comes from its capability in data movement reduction.

### 6.9. Graph Decoding With Nengo

In this section, we demonstrate GrapHD memory decoding using the Nengo SPA module to simulate how our model can work with Neuromorphic hardware and support existing models that try to make more brain-like models of cognition and reasoning. A key example where our model can be applied is SPAUN (Stewart et al., 2012), which is a large-scale cognitive model of the brain. SPAUN consists of about 2.3 million spiking neurons which are used to run various tasks like addition, digit recognition, and question answering without requiring any rewiring of the neurons. SPAUN represents information using Holographic Reduced Representation (HRR) (DuBois and Phillips, 2017), where the hypervectors are unit real vectors, and the binding is done using circular convolution. Our model for storing graph memory can be used to better represent associated information and correlated memory events in graph-based format and also decoded using the algorithms in this paper.

The implementation of GrapHD uses the HRR encoding that comes with the Nengo SPA module. The module implements HRR operations like binding, bundling and similarity using a Spiking Neural Network architecture. Our implementation contains an encoding module and decoding module. First, we generate random *D* = 64 dimensional vectors for each of the nodes. The encoding module then constructs the Graph memory of the graph based on the algorithm in Section 3. This is done using the binding and bundling operations implemented in Nengo SPA. The decoding module requires additional steps of first unbinding the memory vector with all possible node vectors, and then checking the similarity of the result with all other nodes. Based on the thresholding process, it is then decided whether an edge between two nodes exist. Calculating the similarity with all the nodes is done automatically by Nengo where it checks the similarity between all the semantic pointers in the vocabulary of the model. The main step is in unbinding the memory vector with all the nodes, which is done by calculating the unbinding of a query vector and memory vector. The query vector is chosen to cycle through all the node vector over a period of 0.5*s*, and then Nengo calculated the similarity of the result with all the node vectors as a function of time.

**Figure 14:**
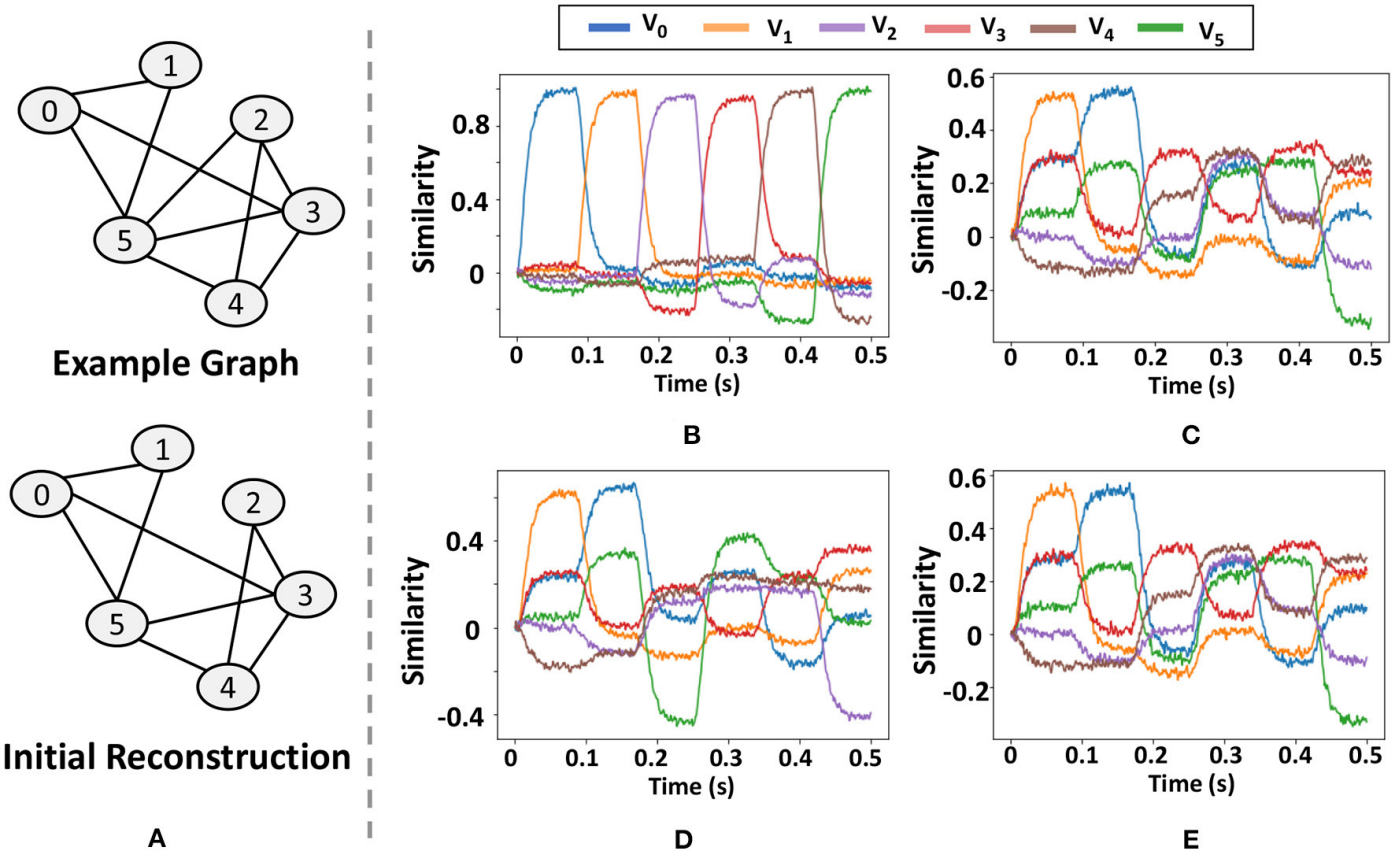
Results of the graph decoding process on neuromorphic hardware. **(A)** graph structure and the initial reconstructed graph, **(B)** similarity of the query vector with each of the nodes, **(C)** similarity with each of the node vector with graph memory of the model, **(D,E)** output of the graph decoding process in the first **(D)** and second iterations **(E)**.

As Figure 14A shows, we use a graph with six vertices and ten edges and demonstrate at each step how the neuromorphic model of GrapHD decodes the graph. We decode the memory at each iteration by sending a query signal for a total of 0.5 s which sequentially changes value from the SPA representing ${\vec{V}}_{0}$ to ${\vec{V}}_{5}$. From the output similarity at each of these time frames with the rest of the vertices, we can understand whether a connection between two nodes exists. For example, consider Figure 14C, which is the first iteration of the decoding process. To find whether an edge between node 2 and node 3, we look at the time of 0.21 s, when the query has the vector representing node 2 and then find the similarity of the line representing node 3 (Red). This similarity is about 0.3, which is greater than the threshold value (chosen to be 0.1).

In Figure 14, we show the results of the Graph decoding process. The figures show the similarity of the semantic pointer with each of the vectors representing all the nodes. In Figure 14B, we show the similarity of the query vector with each of the nodes as a function of time. It keeps cycling between all the nodes once within one cycle of 0.5 s. In Figure 14C, we show the Graph memory of the model and its similarity with each of the node vectors when we feed in the query vector to calculate the similarity. In Figures 14D,E, we show the output of the graph decoding process in the first and second iterations. We find that the output graph in the first iteration has an edge missing, but then it finds this edge and accurately decodes the graph in the second iteration.

Besides SPAUN, as vector symbolic architecture, GrapHD has full compatibility with the new Intel neuromorphic framework, i.e., LAVA. This further shows the capability of GrapHD to be used as neuromorphic computing framework.

## 7. Conclusion

This paper defines a brain-inspired system, called GrapHD, that better represents HDC memorization capability in terms of a graph of relations. We introduce, GrapHD, graph-based hyperdimensional memorization that represents information into high-dimensional space and enables reasoning. GrapHD defines an encoding method that represents complex graph-based data structure into high-dimensional space. Our encoder spreads the information of all nodes and edges across into a full holistic representation so that no component is more responsible for storing any piece of information than another. Then, GrapHD defines several important cognitive functionalities over the encoded memory graph. These operations include memory reconstruction, information retrieval, graph matching, and shortest path.

## Data Availability Statement

The original contributions presented in the study are publicly available. This data can be found here through Microsoft COCO dataset: https://cocodataset.org/#home.

## Author Contributions

PP and MI conceived the research. PP, HA, AZ, FI, MHN, TG, and MI conducted the research and analyzed the data. PP, HA, FI, TG, and MI wrote the manuscript. All authors reviewed the manuscript and agreed on the contents of the paper.

## Funding

This work was supported in part by National Science Foundation (NSF) #2127780 and #2019511, Semiconductor Research Corporation (SRC) Task No. 2988.001, Department of the Navy, Office of Naval Research, Grant #N00014-21-1-2225 and #N00014-22-1-2067, Air Force Office of Scientific Research, the Louisiana Board of Regents Support Fund #LEQSF(2020-23)-RD-A-26, and a generous gift from Cisco.

## Conflict of Interest

The authors declare that the research was conducted in the absence of any commercial or financial relationships that could be construed as a potential conflict of interest.

## Publisher's Note

All claims expressed in this article are solely those of the authors and do not necessarily represent those of their affiliated organizations, or those of the publisher, the editors and the reviewers. Any product that may be evaluated in this article, or claim that may be made by its manufacturer, is not guaranteed or endorsed by the publisher.
